# Cost-Effectiveness Analysis of Direct-Acting Antiviral Agents for Occupational Hepatitis C Infections in Germany

**DOI:** 10.3390/ijerph17020440

**Published:** 2020-01-09

**Authors:** Melanie Runge, Magdalene Krensel, Claudia Westermann, Dominik Bindl, Klaus Nagels, Matthias Augustin, Albert Nienhaus

**Affiliations:** 1 Competence Centre for Epidemiology and Health Services Research for Healthcare Professionals (CVcare), University Medical Centre Hamburg-Eppendorf (UKE), 20246 Hamburg, Germany; melanierunge1994@gmail.com (M.R.); albert.nienhaus@bgw-online.de (A.N.); 2 Institute for Health Services Research in Dermatology and Nursing (IVDP), University Medical Centre Hamburg-Eppendorf (UKE), 20246 Hamburg, Germany; m.krensel@uke.de (M.K.); m.augustin@uke.de (M.A.); 3 Chair of Healthcare Management and Health Services Research, University of Bayreuth, 95445 Bayreuth, Germany; dominik.bindl@uni-bayreuth.de (D.B.); klaus.nagels@uni-bayreuth.de (K.N.); 4 Department of Occupational Medicine, Hazardous Substances and Public Health, Institution for Statutory Accident Insurance and Prevention in the Health and Welfare Services (BGW), 22089 Hamburg, Germany

**Keywords:** hepatitis C, cost-effectiveness analysis, interferon-free therapies, direct-acting antiviral agents, occupational disease

## Abstract

Around 1% of the world’s population is infected with hepatitis C. The introduction of new direct-acting antiviral agents (DAAs) in 2014 has substantially improved hepatitis C treatment outcomes. Our objective was to evaluate the long-term cost effectiveness of DAAs in health care personnel (HP) with confirmed occupational diseases in Germany. A standardised database from a German statutory accident insurance was used to analyse the cost-effectiveness ratio for the DAA regimen in comparison with interferon-based triple therapies. Taking account of the clinical progression of the disease, a Markov model was applied to perform a base case analysis for a period of 20 years. The robustness of the results was determined using a univariate deterministic sensitivity analysis. The results show that treatment with DAAs is more expensive, but also more effective than triple therapies. The model also revealed that the loss of 3.23 life years can be averted per patient over the 20 years. Compared to triple therapies, DAA treatment leads to a higher sustained virologic response (SVR). Although this results in a decrease of costs in the long term, e.g., pension payments, DAA therapy will cause greater expense in the future due to the high costs of the drugs.

## 1. Introduction

Around 1% of the world’s population is infected with the hepatitis C virus (HCV) [1]. Hepatitis C is among the most widespread infectious diseases in the world and is associated with a high level of morbidity and an elevated risk of developing hepatocellular carcinoma [2]. Extrahepatic manifestations may also develop. Since the infection usually occurs with non-specific symptoms, it often remains undetected [2]. Due to the potentially serious course of the disease and the associated costs, a successful treatment is not only in the interest of those affected, but also in the interest of the social insurance institutions [3]. Until 2011, dual therapy using pegylated interferon and ribavirin (PEG-IFN and RBV) was the standard course of treatment. Despite prolonged treatment periods and pronounced side effects, only approximately 40–50% of patients were cured [4]. “Cure” in this context refers to a sustained virologic response (SVR) reflected in the absence of any identifiable HCV ribonucleic acid (HCV-RNA) twelve weeks after the end of treatment (SVR12) [5]. The introduction of first-generation direct-acting antiviral agents (DAAs) in 2011 resulted in an improvement in the SVR12 rate. Triple therapy with the DAA telaprevir (TVR) or boceprevir (BOC) in combination with PEG-IFN and RBV resulted in SVR12 rates of up to 75% with HCV genotype 1 infections, but with the known side effects of PEG-IFN/RBV therapies [6]. The second-generation DAAs substantially improved treatment of chronic hepatitis C (CHC) [7]. These DAAs, introduced in 2014, provided effective interferon-free therapy combinations for all genotypes. Publications report SVR12 rates of over 90% and above, including patients with therapy experience and with advanced stages of disease [8,9]. These orally administered drugs have shorter treatment periods (8, 12, or 24 weeks) and improved tolerance compared to interferon-based therapies. Due to these advantages the current guidelines regarding CHC treatment recommend a DAA therapy for all CHC patients in Germany—regardless of the stage of the disease and the cost of treatment [10,11,12]. Hepatitis C is among the most common infectious diseases to be recognised as an occupational disease by the Institution for Statutory Accident Insurance and Prevention in the Health and Welfare Services (BGW) [13]. Contact with infected patients during work elevates the risk of infection for health care personnel (HP). In particular, injuries with sharp or pointed objects, which are among the most frequently reported occupational accidents in Germany, increase the risk of infection [14,15]. The aim of the study is to determine the cost-effectiveness ratio of second-generation DAAs compared to interferon-based triple therapies based on data from the BGW. It examines the associated treatment results and costs and the impact of these in the case of HP. Modelling is used to simulate the impact of DAA treatment on future changes in outcomes and costs.

## 2. Materials and Methods

We used routine data records of occupational disease from the German Statutory Accident Insurance in the Health and Welfare Services (BGW) to analyse treatment outcomes and associated costs arising from DAA treatment versus those of interferon-based therapy. This study was performed in line with the “Consensus German Reporting Standard for Secondary Data Analyses (STROSA)” [16] and with the “Consolidated Health Economic Evaluation Reporting Standards (CHEERS) statement” (Supplementary Table S1) [17]. 

### 2.1. Data Sources

The occupational disease routine data analysis was based on insured HP for whom an occupational CHC infection was recognised in Germany between 2000 and 2017. The data records contain sociodemographic characteristics as well as information on compensation benefits and the degree of reduced work ability (RWA). To analyse the incremental cost-effectiveness ratio (ICER) for DAA treatments compared to that of interferon-based therapy, additional treatment-related data such as the SVR12 rate and information on whether patients were treated with DAAs or not were taken into account. Based on standardised records, Westermann et al. [9] reported data of insured HP with occupational CHC infection, who applied for a DAA therapy to BGW. To identify HP with DAA treatment, the data sets of the overall cohort and the DAA records were linked using the reference variables “reference ID” by a confidential records handler within BGW. HP with an application for DAA treatment and with information about SVR12, cirrhosis, and RWA were included in the DAA data set for performing the Markov model. The data protection concept was reviewed by the data protection officer of BGW. The external group of researchers in Hamburg performing the analysis received anonymised data from the occupational disease cases under study. In accordance with the Professional Code for Physicians in Hamburg (Art. 15, 1) and the Chamber of Legislation for Medical Professions in the Federal State of Hamburg (HmbKGH), the analysis of anonymised data is exempt from obtaining advice on questions of professional ethics and professional conduct from an ethics committee.

### 2.2. Retrospective Cost Study

In the first part of our study, we conducted a retrospective analysis of expenditures arising from medical benefits and pension expenditure on pensions and payments in HP affected by HCV from 2000 to 2017. 

### 2.3. Prospective Cost-Effectiveness Analysis (CEA)

#### 2.3.1. Assumptions for the SVR12 Rates

In the second and last part of this evaluation, we carried out a prospective study using results from retrospective analysis. Within this analysis, the SVR12 rate was used as a clinical parameter for measuring the medical effectiveness of antiviral treatments on HCV infections. This approach is in line with the decision of the Joint Federal Committee (GBA) and the German Institute for Quality and Efficiency in Health Care (IQWIG). In the absence of information on the achievement of SVR12, the corresponding cost and performance curves were considered and one or no SVR12 was assumed. Most of the study cohort had an HCV genotype 1 infection and prior experience with treatment. The main DAA regimen used was sofosbuvir (SOF) combined with ledipasvir (LDV) (±RBV) [9]. The model assumptions were made under these conditions. For the model, the SVR rates were determined using the available literature [18,19,20]. As a basis, SVR12 rates of 95.4% were assumed for non-cirrhotic patients with therapy experience and 96.1% for cirrhotic patients with therapy experience [18]. For interferon-based triple therapy (boceprevir, BOC + PEG-IFN + RBV), SVR12 rates of 64.4% were assumed for non-cirrhotic patients with therapy experience and 35.3% for cirrhotic patients with therapy experience [18,21,22]. 

#### 2.3.2. Assumptions for Health State Costs

Information on HP insured by the BGW was used to quantify the effect of DAA treatment on the future development of costs. The direct medical costs for the defined disease statuses were extrapolated over the year. Services for inpatient and outpatient treatments, diagnostic examinations, laboratory testing, drugs, and other therapeutic procedures were taken into account. The indirect costs caused by the RWA were recorded in the form of pension payments. The utilisation of health care services and the average cost points per patient were applied. Stahmeyer et al. [18] provided the reference results for determining the costs of the disease statuses “decompensated cirrhosis”, “hepatocellular carcinoma”, and “liver transplantation”. These cost parameters include drugs, diagnostic procedures, and treatment, including treatment of side effects [18]. The costs applied for diagnostic procedures are based on Stahmeyer et al. [23]. The costs per disease statuses were derived from published literature [24,25,26]. Table 1 shows the values applied for the model. 

#### 2.3.3. Model Structure

**Figure 1:**
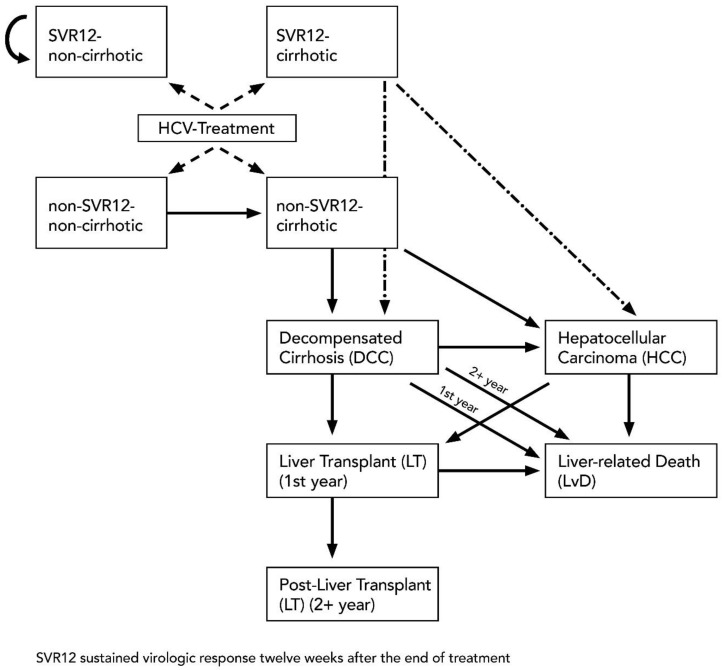
Markov modelling for cost-effectiveness analysis.

To determine the long-term cost effectiveness of interferon-free drugs, a Markov model was developed (Figure 1). A Markov model was used to predict the clinical course of hepatitis C in the case of unsuccessful therapy and the associated long-term costs for the BGW. The model takes into account the costs incurred by patients in the year subsequent to the treatment. This allowed direct costs associated with medical treatment and indirect costs such as pension benefits to be allocated to the stages. The majority of HP with recognised CHC had a HCV genotype 1 infection and experienced previous treatment [9]; the model has been developed for this specific group of patients for a time line of 20 years with a cycle length of one year. This assumption reflects the slow progression rate of chronic diseases [3,18,27]. The model takes into account the clinical progression of a CHC infection. Progress is denoted by various disease statuses (non-cirrhotic, cirrhotic, decompensated cirrhosis, hepatocellular carcinoma, (post-)liver transplantation, liver-related death).

Given the lack of clinical information in the data set, differentiation was performed based on disease status (e.g., cirrhosis). In line with Westermann and colleagues [9], we assumed that cirrhosis was present where RWA was at least 50%. To verify the correlation between an RWA ≥50% and the presence of cirrhosis in the DAA study cohort, the Pearson correlation coefficient was used. The significance of the correlations was tested using Fisher’s exact test. 

Patients were entered into the Markov model at the statuses “SVR12—non-cirrhotic”, “SVR12—cirrhotic”, “non-SVR12—cirrhotic”, or “non-SVR12—non-cirrhotic”. In the BGW study cohort, approximately 17.2% of patients were identified as being in a cirrhotic status before treatment and therefore entered the Markov model at this stage. 

In non-cirrhotic patients, the disease progression is stopped when SVR12 is achieved. Patients with cirrhosis may still develop decompensated cirrhosis (DCC) or hepatocellular carcinoma (HCC) despite reaching SVR12, but the likelihood of this is significantly lower compared to patients without SVR12 [28]. The transition probabilities are derived from available literature and shown in Table 2. According to the recommendations of IQWIG, an annual discount rate of 3% was applied [29].

To enable a comparison of the benefits and costs of DAA therapies with alternative therapies from the past, the model was run using the same cohort treated with triple therapy. Here, relevant model assumptions such as SVR12 and cost parameters were adjusted accordingly. Because cost calculation was not possible based on the data, we assumed the cost of triple therapy to be approximately 50% of the cost of DAA therapy. Information from literature supports this assumption [27,43]. The simulation was carried out using Excel Methods from the manual “Cost Effectiveness Modelling for Health Technology Assessment” [44]. For the long-term cost calculation in this study, costs of each health status were offset to the probability of a health status occurring. The model was programmed for both therapies using Microsoft Excel Version 3.0, Microsoft Corporation, Redmond, WA, USA (released 2013).

#### 2.3.4. Liver-Related Mortality Rate

The model also incorporated life years lost as an additional outcome. This parameter was calculated by offsetting the distribution values of the disease stage “liver-related death” (LvD) against the life years lost. This resulted in life years lost from the total longevity of a patient in “LvD” status over the observation period of 20 years. 

#### 2.3.5. Sensitivity Analysis

To evaluate the robustness of the model, a univariate deterministic sensitivity analysis (DSA) was performed. The listed transition probabilities were varied via their minimum and maximum values, while the outcomes, direct costs of the disease status were varied by ±25%. For changes in pension benefits, indirect costs were also varied by ±25%. Based on a high dynamic in the costs for DAA drugs, the individual cost components of medical care were varied by ±50%. Additionally, the effect of cirrhosis on the result was determined by assuming that all patients entering the model were allocable to the (non-)cirrhotic disease status.

The statistical analyses were performed using IBM SPSS Statistics for Windows, Version 24.0, IBM Corp, Armonk, NY, USA (released 2014) and Microsoft Excel Version 3.0, Microsoft Corporation, Redmond, WA, USA (released 2013).

## 3. Results

### 3.1. Retrospective Data Analysis

**Figure 2:**
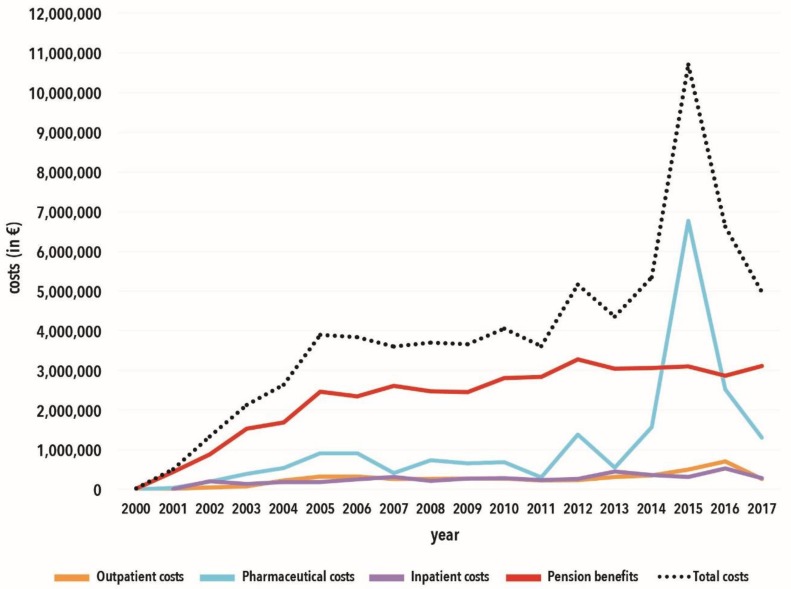
Cost trends of medical services and pension benefits of the Institution for Statutory Accident Insurance and Prevention in the Health and Welfare Services (BGW).

We analysed data from 820 HP affected by an occupational HCV infection between 2000 and 2017. The expenses for pensions and medical benefits of HP are presented in Figure 2. Compensation payments for medical treatment (outpatient and inpatient treatments and drugs) and pensions have developed differently over the period under review. Annual expenses rose from €29,000 to €3.9 million from 2000 to 2005, remaining at an annual expense of approximately €4 million between 2005 and 2011 before peaking at around €11 million in 2015. In all years except 2014 and 2015, payments for pensions dominated the cost of compensation. With the introduction of the second-generation DAAs in 2014, there was also an increase in costs for the BGW. This was due to the fact that the costs for drug therapy were significantly higher compared to those for interferon-based therapy. In addition, many cases from previous years in which treatment was unsuccessful were again treated with DAAs, especially when DAAs had just been introduced. The total annual expenditure and the costs for medical treatments have declined since 2015. In 2016, the cost for drugs alone went below the annual cost for pension benefits.

### 3.2. Prospective CEA—Markov Model

#### 3.2.1. Description of Data Set

**Figure 3:**
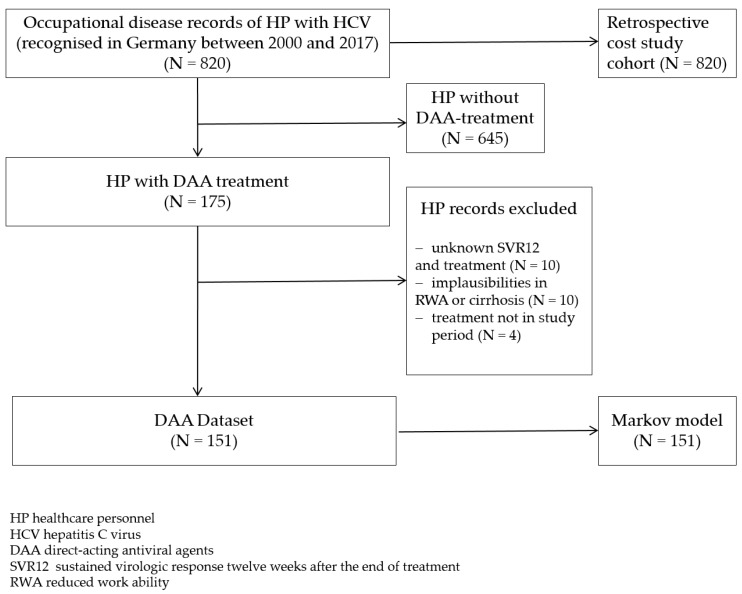
Study overview. DAA—direct-acting antiviral agent.

For the Markov model, 151 insured persons out of the 820 cases in the occupational disease records were identified as DAA patients (Figure 3). 

Sociodemographic characteristics of patients are presented in Table 3. Of the DAA patients, 78% were women, and most were at least 50 years of age when treatment commenced. Among the DAA patients, the majority were employed in medical/nursing professions, with over 50% serving as nurses, 25% as medical assistants, and 15% as physicians. The largest share of insured HP worked in clinics (around 47%), in general human medicine (26%), and dental medicine (9%) and in nursing (9%). A documented RWA of ≥50% applied to 23% of the HP. The correlation coefficient between an RWA of ≥50% and cirrhosis was positive (*r* = 0.83, *p* < 0.001). There was a large and statistically significant linear relationship between an RWA of ≥50% and the presence of cirrhosis in the DAA collective before treatment commenced. After DAA treatment, 93% of the insured persons reached SVR12. Taking into account the manually reviewed cost and outcomes of patients with missing information on the response rates, the SVR12 resulted in 97%. The benefit of DAA treatment was apparent in the high recovery rates, the low number of reports of adverse side effects in the study cohort, and improvement in the RWA (Table 3). After treatment with the DAAs, an RWA of <50% was documented for 82%, whereas this value was 77% before treatment.

#### 3.2.2. 20-Year Prediction

Based on the Markov cohort simulation, the treatment results for each HP over the next 20 years can be predicted with a total cost of €206,184 for DAA patients with therapy experience (Supplementary Figures S1 and S2). By comparison, the costs over 20 years for triple therapy come to €171,017. Therefore, total costs within this period are €35,167 higher for patients treated with DAAs (Supplementary Figures S3 and S4).

#### 3.2.3. Cost Effectiveness

As shown above, treatment with DAAs results in better outcomes than triple therapies, but also causes higher costs. In comparison with triple therapy, DAA treatment results in an ICER of €766.19 per SVR12 percentage point (Table 4). 

DAA therapy proved to be an effective treatment strategy. Compared to triple therapy, the higher SVR12 rates can help to prevent liver cirrhosis and other liver disorders or manifestations. This study observed an average gain of 3.23 life years over the 20-year horizon for each patient in the model. 

#### 3.2.4. Sensitivity Analysis

**Figure 4:**
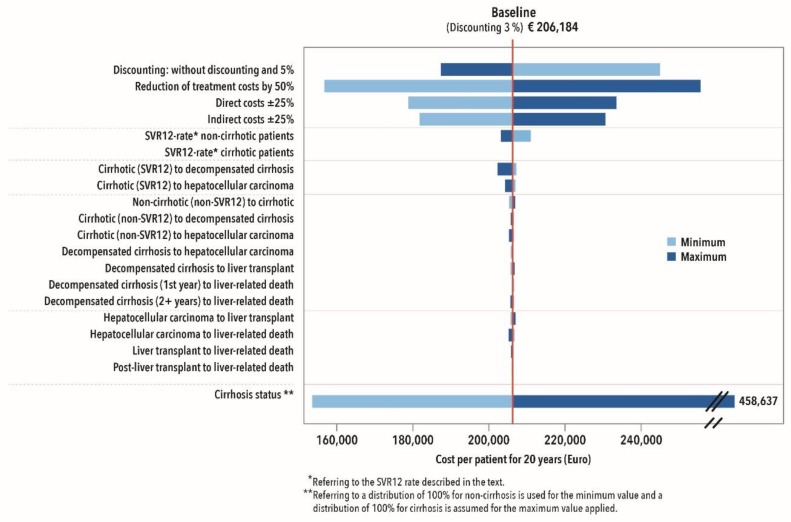
Deterministic sensitivity analysis of DAA therapies.

**Figure 5:**
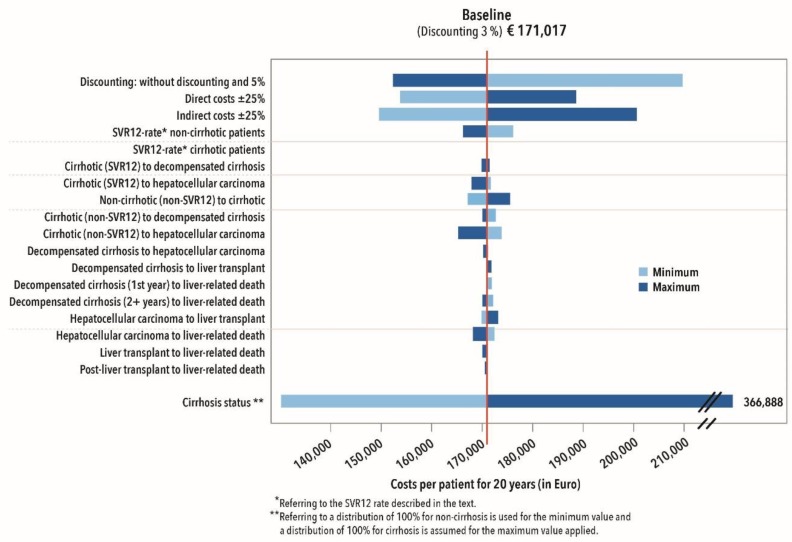
Deterministic sensitivity analysis of interferon-based triple therapies.

In a univariate DSA, the variables with the greatest influence on the variability of the result were identified. The analyses were performed separately for triple and DAA therapies. With both treatment regimens, the variations in costs and SVR12 rates and the fibrosis/cirrhosis status had the greatest impact on the results under the Markov model (Figure 4 and Figure 5).

Assuming that pension payments will fall by 25% over the following years due to the higher recovery rates, this would also have a strong impact on the total costs for the BGW. Where costs increase, the result reacts sensitively. The fibrosis/cirrhosis status of the patient also has a considerable impact on total cost. Treatment of insured persons without cirrhosis correlates with a cost reduction. Varying transition probabilities in the model only have a minor effect on the overall result (Figure 4). 

Comparable results were also found in relation to treatment with interferon-based triple therapy (Figure 5). However, varying transition probabilities in the interferon-based therapy model have a greater impact on the overall result.

## 4. Discussion

Based on a group of insured HP, the cost effectiveness of DAA treatment regimens could be analysed for the first time in Germany, taking pension payments into account. Our results show cost effectiveness for these treatments assuming willingness to pay of an additional €35,167 per patient over 20 years. This result is comparable with findings from available literature [18]. For therapy with DAA treatment regimens, an ICER of €766.19 per added SVR12 rate percentage point was identified. This means that it takes €766.19 to generate an extra percentage point for the SVR12 as a result of the switch from triple therapy to DAAs. DAA therapy proved to be an effective treatment strategy. After treatment with the DAAs, an RWA of <50% was documented for 82%, whereas this value was 77% before treatment. In the study cohort, treatment with DAAs was also associated with a reduction in life years lost. This result is consistent with information from available literature [45]. Prevention of late-stage consequences of HCV infection, a reduction in the burden involved in such a disease, and an improvement in quality of life are significant added-value parameters afforded by DAA therapies [45,46]. Because cirrhosis correlates heavily with the liver-related mortality rate, early application of the therapy is all that much more important [45]. 

Several studies have analysed the cost effectiveness of DAAs in an international comparison, however for Germany there are as yet only a small number. In our analysis, the SVR12 rate was used as a clinical parameter for measuring the medical effectiveness of treatments on HCV infections. The benefit value was therefore applied in additional SVR12 percentage points. This approach is in line with the resolution of the Joint Federal Committee (GBA) and the German Institute for Quality and Efficiency in Health Care (IQWIG), thus the results of this CEA can be applied directly in clinical practice. As in our study, the benefits were measured using the SVR12 rate in a CEA from Germany [43]; Giesel et al. calculated an ICER of €1560.13 per additional SVR12 percentage point. In contrast to the present study, the Markov model was developed for treatment-naive patients treated with the DAAs SOF and/or simeprevir (SMV). The ICER for Gissel et al. [43] is more than twice our value, which results from their comparison of treatment using SOF + RBV for 24 weeks and SOF + SMV ± RBV for 12 weeks. 

In an international context, the benefit value was applied in the form of the quality-adjusted life years (QALYs) in the CEA. QALYs are stated as the product of the further life expectancy with a disease/treatment success and a value for the quality of life. The SVR itself only describes the cure rate, without considering the patient’s quality of life. The QALYs benefit measurement therefore has a different calculation approach, which makes it difficult to compare the cost–benefit ratio of our study with international studies that use QALYs as a benefit measurement. The identified ICER values are therefore not comparable with the value determined here, but they do allow the results of this study to be placed into some kind of context. The study by Stahmeyer et al. [18] analysed the cost effectiveness of DAA therapy regimens in Germany. The model calculated the life cycle costs and QALYs for SOF with LDV compared to alternative treatments. The analyses show that the application of SOF in combination with LDV for patients with treatment experience compared to other alternative therapies was a compelling proposition with an ICER of €26,426/QALY and a willingness to pay of €30,000. The present model assumes a cost effectiveness with a willingness to pay of €35,167 per patient. This value is higher than that of Stahmeyer et al. [18]. However, it must be considered that the assumed value related to treated patients, not to QALYs. In their efficiency analysis, Mühlbacher and Sadler [47] studied the DAA therapy options currently available on the market in Germany. In a comparison between the various DAA therapy regimens and the interferon-based triple therapy, SOF with LDV and ombitasvir (OBV) + paritaprevir (PTV) + ritonavir (RTV) + dasabuvir (DSV) ± RBV were the most cost-efficient alternatives. The cost effectiveness of SOF + LDV as presented in the Mühlbacher and Sadler study [47] supports the cost-effectiveness findings of the present study regarding DAA treatment compared to triple therapy. On the international level, several studies have analysed the cost effectiveness of DAAs. Younossi et al. [3] came to the conclusion that the treatment of all HCV patients with interferon-free therapy would be the cheapest strategy with an ICER of $15,709/QALY for treatment-naive patients. For the treatment of HCV genotype 1, triple therapy by disease status and independent of disease status was compared with interferon-free DAA therapy by disease status and without. Chhatwal et al. [48] concur with these findings in their study on the cost -effectiveness of SOF with LDV in a study cohort comprising both treatment-naive patients and patients with treatment experience from the USA. SOF-based therapies exhibited cost effectiveness with an ICER of $55,400/QALY. Zhao et al. conducted a study with comparable results to analyse the cost–benefit relationship of second-generation DAAs. The study compared a triple therapy against DAA regimens SOF + LDV, SOF + SMV, OBV + PTV + RTV + DSV ± RBV. For all DAA treatments, a cost effectiveness with an ICER of $50,828/QALY was found in a comparison with interferon-based treatments [27]. Internationally, the majority of the studies stated cost-effectiveness figures regarding treatment with DAAs, in particular treatments using SOF with LDV, compared to interferon-based therapies. In a comparison of CEAs for DAAs, the underlying data must be considered. This study also takes into account indirect costs such as pension payments. The comparability of the results is therefore limited. In the data used in the present study, only occupational disease reports for HP from non-governmental health institutions in Germany are taken into account. Health care systems, supply structures, and pay systems may vary from insurer to insurer and country to country. 

### Limitations and Strengths

Restrictions that generally apply for secondary data also apply to the data from the BGW in this analysis. [9]. The lack of disease-specific information is a particular limitation of the model. The data on the effectiveness of interferon-based and DAA therapies used in the model are based on the results of a literature review. These are only applicable to clinical practice to a limited extent. Data from the occupational disease database confirm the high SVR12 rates for DAA treatment, amounting to 97%. In contrast, the SVR12 rates according to Stahmeyer et al. [18] used in the model for the DAA regimen are 95.4% or 96.1%, depending on cirrhosis status, so the effectiveness is understated somewhat. The use of cirrhosis as a predictor for significantly reduced success rates (SVR12) is not reflected in the model. While no significant difference between SVR12 rates in relation to DAA treatment and cirrhosis is apparent in Stahmeyer et al. [18], a previous study by Westermann et al. [9] found a significant reduction in SVR12 in cirrhotic patients. The assumed recovery rates in the case of triple therapy (BOC + PEG-IFN + RBV) are based on the study by Stahmeyer et al. [18]. The benefits of DAA therapy would probably be greater if a comparison was done with PEG-IFN + RBV therapy instead of triple therapy. In patients without available information regarding the cirrhosis status, we assumed a correlation with RWA levels ≥50%, which was confirmed to be positive to a statistically significant degree (*r* = 0.83, *p* < 0.001).

These data, based on BGW benefits records, only allow average direct costs to be calculated for the stages “SVR12—non-cirrhotic”, “SVR12—cirrhotic”, “non-SVR12—non-cirrhotic”, and “non-SVR12—cirrhotic”. Cost values expressed by Stahmeyer et al. [18] were used for all remaining stages in the model. Stahmeyer et al. [18] based their work on findings by Stahmeyer et al. [23], Siebert et al. [24], Stahmeyer et al. [25], and Wasem et al. [26]. These used values apply to the circumstances of a statutory health insurance. Expenditures specific to accident insurance such as costs for reports and expert appraisals have not been taken into account, whereas pension payments were obtained from occupational disease records. This means that the average cost for the BGW in the “decompensated cirrhosis” and stages of progression from there may potentially be understated. This CEA is based on real-world data from insured HP of the BGW, reflecting the distribution in the population studied. It was expected that the insured HP would mainly be women (typical for the gender distribution in health care professions in Germany) with many years of experience in illness and therapy [49]. Most DAA patients were 50 years and older at the start of treatment. According to Westermann et al. [9], advanced age has no negative influence on successful DAA treatment.

Despite the limitations, the present study allows the benefits and costs of DAA treatments to be examined in connection with statutory accident insurance-financed services. It must be taken into consideration that the costs recorded are not standardised costs, but actual costs incurred for the study cohort. The costs of the 20-year projection relate to this study cohort comprising patients with therapy experience. Due to the declining incidence of HCV infections among HP, it is expected that the overall cost for treating occupational HCV infections will also fall for statutory accident insurance [13]. Following an increase in the number of patients treated with DAAs in 2015, this figure declined again and is now largely stable [13]. The retrospective cost analysis in the present study supports this assumption. The overall cost for occupational CHC cases has fallen in recent years. Kruger et al. [8] estimated in a real-world setting study using data from the German Hepatitis C Registry that costs per SVR12 for second-generation DAA treatments are comparable to those for first-generation DAAs, due to the fact that the costs for the currently used treatment regimens have declined. Additionally, the high SVR12 rates involved in DAA treatment resulted in a lower RWA for most insured persons in our study. In the long term, this will be positively reflected in the cost structure, especially in terms of the expenditure for pension benefits. The results of the sensitivity analysis show that prompt administration of therapy is desirable for cost reasons. Treatment with interferon-free therapies reduces the number of patients with advanced liver diseases and increases life expectancy. Newly introduced treatment regimens such as Epclusa (SOF + velpatasvir), Zepatier (elbasvir + grazoprevir), or Maviret (glecaprevir + pibrentasvir) allow lower-cost alternatives to be offered for newly infected patients than DAA regimens with SOF [50,51]. In particular, the very well tolerated ribavirin-free DAA regimen pibrentasvir/glecaprevir achieves an overall SVR12 rate of 98% with a short treatment duration and a high barrier to resistance. It is the only pangenotypic therapy regimen for patients with severe-to-terminal renal dysfunction, including dialysis patients, and is also well suited for patients following liver transplantation [51,52]. When the patent for the costly DAA regimen (e.g., SOF) expires, cheaper generic drugs will be available in future to treat CHC. To regulate treatment costs, it is advisable to prioritise individual DAA treatment regimens based on their cost effectiveness. Alongside the economic perspective, ethical concerns are also of importance.

## 5. Conclusions

The use of DAA therapies has fundamentally changed the treatment of CHC infections. However, the high success rates (SVR12) do come at higher costs compared to triple therapies. The results of the CEA show that DAA therapies are more effective, but also more expensive. At the same time, sensitivity analyses show that the statutory accident insurance can save costs if the treatment is carried out promptly. In addition, variations in costs and SVR12 rates also had the greatest impact on the results under the Markov model. In particular, the recently introduced, more favourable DAA regimens promise to improve cost effectiveness through shorter treatment duration and higher success rates. To examine the impact of DAA treatment more comprehensively, prospective studies are required. These should document relevant clinical and patient-related endpoints and analyse the long-term benefit of the new drugs.

## Figures and Tables

**Table 1:** Health state costs, in € per year.

Health State	Base-Case Direct Costs (in €)	Base-Case Indirect Costs (in €)	Range (in %)	Source
Non-cirrhotic (treatment year)	64.518	6.555	±25	o. c.
Cirrhotic (treatment year) entry 2	93.353	20.104	±25	o. c.
Non-cirrhotic (SVR12)	1.595	3.739	±25	o. c.
Cirrhotic (SVR12)	6.734	18.029	±25	o. c.
Non-cirrhotic (non-SVR12)	6.734	4.183	±25	o. c.
Cirrhotic (non-SVR12)	10.171	19.474	±25	o. c.
Decompensated cirrhosis (1st year)	9.768	19.474	±25	[18,23,24,25,26]
Decompensated cirrhosis (2+ years)	9.768	19.474	±25	[18,23,24,25,26]
Hepatocellular carcinoma	24.096	19.474	±25%	[18,23,24,25,26]
Liver Transplant	143.480	19.474	±25%	[18,23,24,25,26]
Post-liver Transplant	20.751	19.474	±25%	[18,23,24,25,26]

o. c. = own calculation; SVR12—sustained virologic response twelve weeks after end of treatment.

**Table 2:** Transition probabilities.

Health State		Base-Case	Upper Range	Lower Range	Source
From:	To:				
Non-cirrhotic	Compensated cirrhosis	0.016	0.008	0.026	[30]
Non-cirrhotic (SVR12)	SVR12 Non-cirrhotic	1	–	–	–
Cirrhotic	Decompensated cirrhosis	0.029	0.010	0.039	[31,32,33,34,35]
Cirrhotic	Hepatocellular carcinoma	0.028	0.010	0.079	[31,32,33,34,35]
Cirrhotic (SVR12)	Decompensated cirrhosis	0.008	0.002	0.036	[36]
Cirrhotic (SVR12)	Hepatocellular carcinoma	0.005	0.002	0.013	[36]
Decompensated cirrhosis	Hepatocellular carcinoma	0.068	0.030	0.083	[37]
Decompensated cirrhosis	Liver transplant	0.023	0.010	0.062	[38,39]
Decompensated cirrhosis (1st year)	Liver-related death	0.182	0.065	0.190	[37]
Decompensated cirrhosis (2+ years)	Liver-related death	0.112	0.065	0.190	[37]
Hepatocellular carcinoma	Liver transplant	0.040	0.000	0.140	[40,41]
Hepatocellular carcinoma	Liver-related death	0.427	0.330	0.860	[32]
Liver Transplant	Liver-related death	0.116	0.060	0.420	[42]
Post-liver Transplant	Liver-related death	0.044	0.024	0.110	[42]

SVR12—sustained virologic response twelve weeks after the end of treatment.

**Table 3:** Description of the study cohort.

Characteristics	*N*	%
**Overall**	151	100%
**Gender**		
Woman	118	78.1%
Men	33	21.9%
**Age group on therapy**		
≤39	3	2.0%
40–49	19	12.6%
50–59	40	26.5%
>60	63	41.7%
Missing values	26	17.2%
**Professional activity**		
Physician	23	15.2%
Nurse	65	43.0%
Medical technical personnel	2	1.3%
Medical assistant	35	24.5%
Geriatric nurse	14	9.3%
Social workers	1	0.7%
Housekeeping	5	3.3%
Administration and others	4	2.6%
**Side effects**		
None	107	70.9%
Headaches, nausea, sleep disorder	25	16.6%
Skin reactions	3	2.0%
Depression, anxiety	3	2.0%
Gastrointestinal disorders	3	2.0%
Others	10	6.6%
**SVR12**		
Yes	140	92.7%
No	5	3.3%
Missing values	6	4.0%
**RWA before therapy with DAAs**		
<50	116	76.82%
≥50	35	23.18%
**RWA after therapy with DAAs**		
<50	124	82.19%
≥50	27	17.81%

SVR12—sustained virologic response twelve weeks after the end of treatment; RWA—reduced work ability.

**Table 4:** Cost effectiveness.

Discounted Costs (in €), SVR12 Rates and ICER (€/SVR12 Percentage Point)			
	Costs (in €)	SVR12 Percentage Point	ICER (€/SVR12 Percentage Point)
**Triple Therapies**	171.017	49.85 *	–
**DAA Therapies**	206.184	95.75 **	€766.19/SVR12 Percentage Point

* Mean of SVR12 rates boceprevir + pegylated interferon + ribavirin non-cirrhotic + cirrhotic [14]; ** Mean of SVR12 rates sofosbuvir + ledipasvir ± ribavirin non-cirrhotic + cirrhotic [14]; ICER—incremental cost-effectiveness ratio.

## Supplementary Materials

The following are available online at https://www.mdpi.com/1660-4601/17/2/440/s1, Table S1: Consolidated Health Economic Evaluation Reporting Standards (CHEERS) checklist, Figure S1: DAA result calculation, Figure S2: DAA therapy cost calculation, Figure S3: Triple therapy result calculation, Figure S4: Triple therapy cost calculation.
