# Supplementary material for: Cost-Effectiveness Analysis of Direct-Acting Antiviral Agents for Occupational Hepatitis C Infections in Germany
Source: Int J Environ Res Public Health. 2020 Jan 9;17(2):440. doi: 10.3390/ijerph17020440 (PMC7013637; doi:10.3390/ijerph17020440)
Supplement: Supplementary file 1 [file ijerph-17-00440-s001.zip › CHEERS checklist_CEA occupational HCV.pdf]

CHEERS checklist—Items to include when reporting economic evaluations of health interventions

Section/item	Item No	Recommendation	Reported on page No/ line No
**Title and abstract**			
Title	1	Identify the study as an economic evaluation or use more specific terms such as "cost-effectiveness analysis", and describe the interventions compared.	page 1, line 22 to 24
Abstract	2	Provide a structured summary of objectives, perspective, setting, methods (including study design and inputs), results (including base case and uncertainty analyses), and conclusions.	page 1, line 20 to 32
**Introduction**			
Background and objectives	3	Provide an explicit statement of the broader context for the study.	page 1, line 37 to page 2, line 63
		Present the study question and its relevance for health policy or practice decisions.	page 2, line 63 to 67
**Methods**			
Target population and subgroups	4	Describe characteristics of the base case population and subgroups analysed, including why they were chosen.	page 2 line 76 to 87;
Setting and location	5	State relevant aspects of the system(s) in which the decision(s) need(s) to be made.	page 2, line 76 to 79
Study perspective	6	Describe the perspective of the study and relate this to the costs being evaluated.	page 2, line 76 to 77; page 4, line 130 to 140
Comparators	7	Describe the interventions or strategies being compared and state why they were chosen.	page 2, line 79-82 page 6, line 160 to 166
Time horizon	8	State the time horizon(s) over which costs and consequences are being evaluated and say why appropriate.	page 4; line 130 to 140; page 6, line 160 to166
Discount rate	9	Report the choice of discount rate(s) used for costs and outcomes and say why appropriate.	page 5, line 157 page 6, line 160 to 167
Choice of health outcomes	10	Describe what outcomes were used as the measure(s) of benefit in the evaluation and their relevance for the type of analysis performed.	page 3, line 100 to 113;
Measurement of effectiveness	11a	*Single study-based estimates:* Describe fully the design features of the single effectiveness study and why the single study was a sufficient source of clinical effectiveness data.	
	11b	*Synthesis-based estimates*: Describe fully the methods used for identification of included studies and synthesis of clinical effectiveness data.	page 2, line 65 to 67; page 2, line 79 to 82; page 3, line 115 to 125
Measurement and valuation of preference based outcomes	12	If applicable, describe the population and methods used to elicit preferences for outcomes.	not applicable
Estimating resources and costs	13a	*Single study-based economic evaluation:*Describe approaches used to estimate resource use associated with the alternative interventions. Describe primary or secondary research methods for valuing each resource item in terms of its unit cost. Describe any	

Section/item	Item No	Recommendation	Reported on page No/ line No
		adjustments made to approximate to opportunity costs.	
	13b	*Model-based economic evaluation:* Describe approaches and data sources used to estimate resource use associated with model health states. Describe primary or secondary research methods for valuing each resource item in terms of its unit cost. Describe any adjustments made to approximate to opportunity costs.	page 3, line 95 to 97; page 3, line 115 to 125; page 4, Table1; page 4, line 130 to 140; page 5; 143 to 157
Currency, price date, and conversion	14	Report the dates of the estimated resource quantities and unit costs. Describe methods for adjusting estimated unit costs to the year of reported costs if necessary. Describe methods for converting costs into a common currency base and the exchange rate.	page 3, line 115 to 125; page 4, Table 1
Choice of model	15	Describe and give reasons for the specific type of decision-analytical model used. Providing a figure to show model structure is strongly recommended.	page 4, line 130 to 135; page 5, figure 1
Assumptions	16	Describe all structural or other assumptions underpinning the decision-analytical model.	page 4, line 130 to 140; page 5, line 144 to 148; page 5, line 149 to 152; page 6, line 153 to 157
Analytical methods	17	Describe all analytical methods supporting the evaluation. This could include methods for dealing with skewed, missing, or censored data; extrapolation methods; methods for pooling data; approaches to validate or make adjustments (such as half cycle corrections) to a model; and methods for handling population heterogeneity and uncertainty.	page 3, line 107 to 108; page 3, line 110 to 112; page 4, line 119 to 124; page 6, 166 to 172
**Results**			
Study parameters	18	Report the values, ranges, references, and, if used, probability distributions for all parameters. Report reasons or sources for distributions used to represent uncertainty where appropriate. Providing a table to show the input values is strongly recommended.	page 7, line 186 to 1197; page 8, line 206 219; page 9, Table 3;
Incremental costs and outcomes	19	For each intervention, report mean values for the main categories of estimated costs and outcomes of interest, as well as mean differences between the comparator groups. If applicable, report incremental cost-effectiveness ratios.	page 9, line 220 to 229; page 9, Table 4; page 9, line 225-229; page 10, table 4; supplement Figures S1, S2,S3, S4
Characterising uncertainty	20a	*Single study-based economic evaluation:*Describe the effects of sampling uncertainty for the estimated incremental cost and incremental effectiveness parameters, together with the impact of methodological assumptions (such as discount rate, study perspective).	
	20b	*Model-based economic evaluation:* Describe the effects on the results of uncertainty for all input parameters, and uncertainty related to the structure of the model and assumptions.	page 10, 240 to 244; page 11, line 247 to 254; page 10, figure 4; page 11; figure 5
Characterising	21	If applicable, report differences in costs, outcomes, or	not applicable

Section/item	Item No	Recommendation	Reported on page No/ line No
heterogeneity		cost-effectiveness that can be explained by variations between subgroups of patients with different baseline characteristics or other observed variability in effects that are not reducible by more information.	
**Discussion**			
Study findings, limitations, generalisability, and current knowledge	22	Summarise key study findings and describe how they support the conclusions reached. Discuss limitations and the generalisability of the findings and how the findings fit with current knowledge.	page 11, line 259 to page 14, line 377
**Other**			
Source of funding	23	Describe how the study was funded and the role of the funder in the identification, design, conduct, and reporting of the analysis. Describe other non-monetary sources of support.	page 14, line 393 to 397
Conflicts of interest	24	Describe any potential for conflict of interest of study contributors in accordance with journal policy. In the absence of a journal policy, we recommend authors comply with International Committee of Medical Journal Editors recommendations.	page 14, line 400

For consistency, the CHEERS statement checklist format is based on the format of the CONSORT statement checklist
