# Supplementary figures and images for: Cost-Effectiveness Analysis of Direct-Acting Antiviral Agents for Occupational Hepatitis C Infections in Germany
Source: Int J Environ Res Public Health. 2020 Jan 9;17(2):440. doi: 10.3390/ijerph17020440 (PMC7013637; doi:10.3390/ijerph17020440)

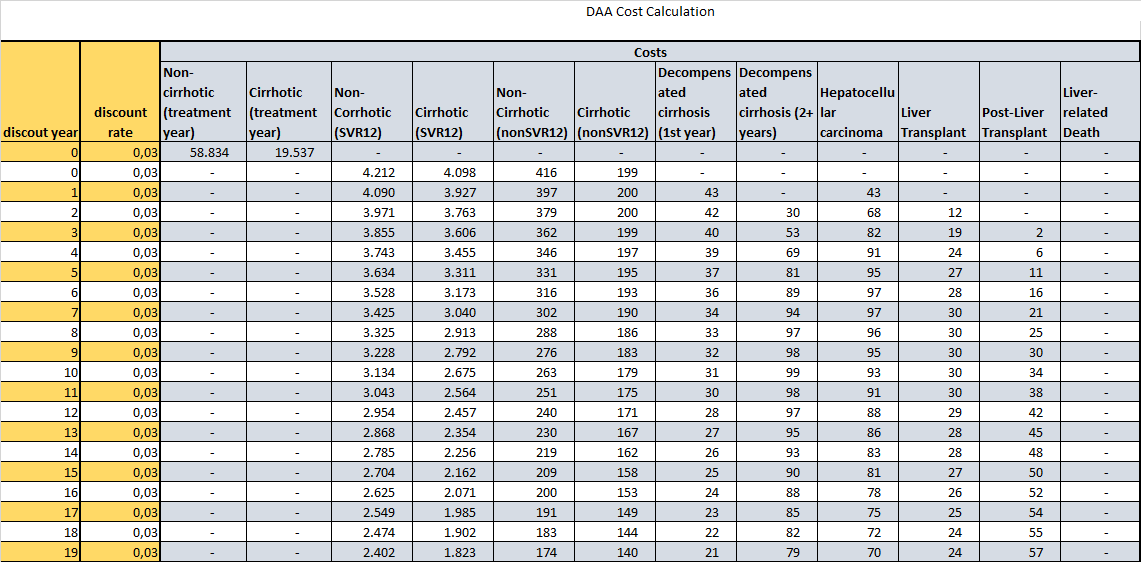

Supplement: Supplementary file 1 [file ijerph-17-00440-s001.zip › DAA Cost Calculation.png]

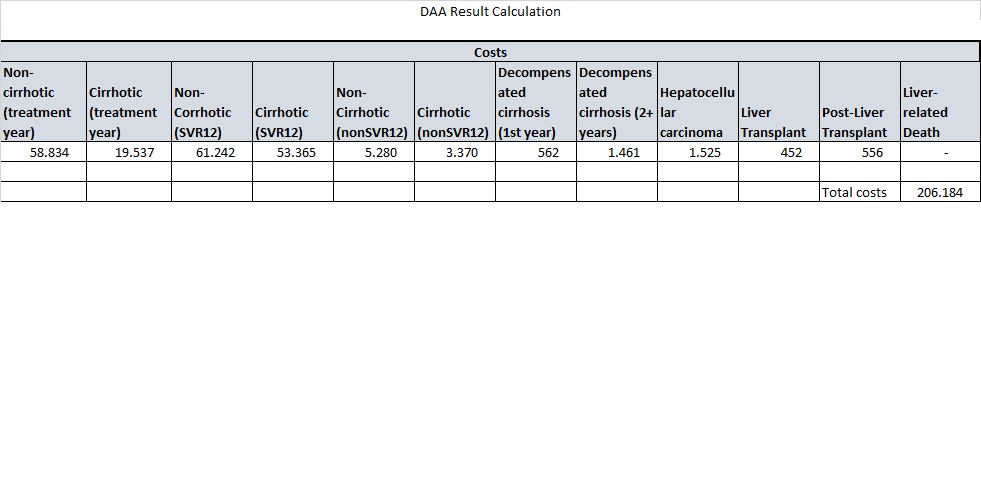

Supplement: Supplementary file 1 [file ijerph-17-00440-s001.zip › DAA Result Calculation.png]

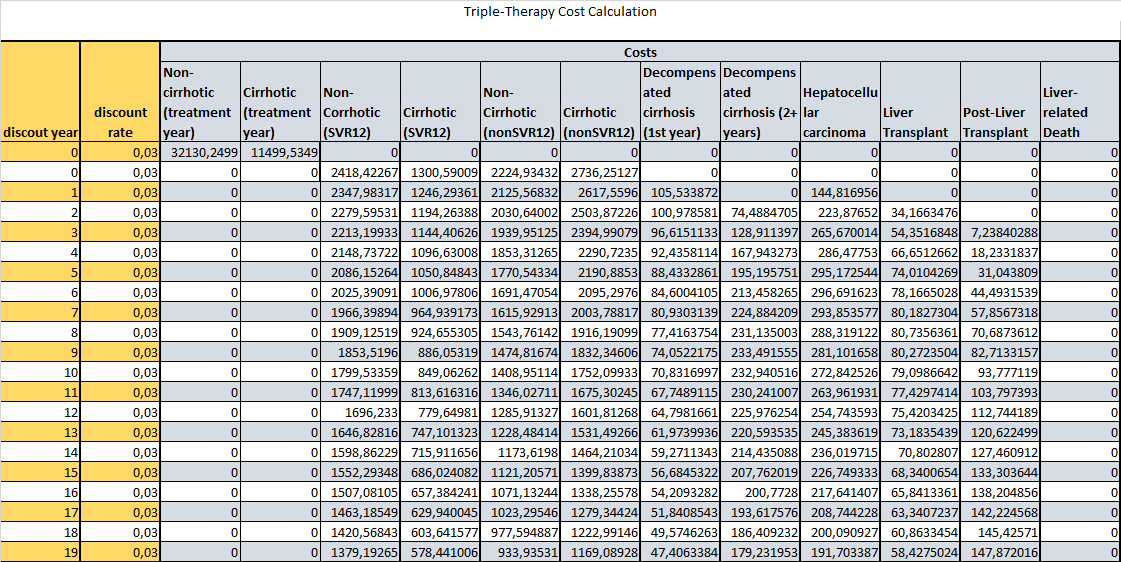

Supplement: Supplementary file 1 [file ijerph-17-00440-s001.zip › Triple Cost Calculation.png]

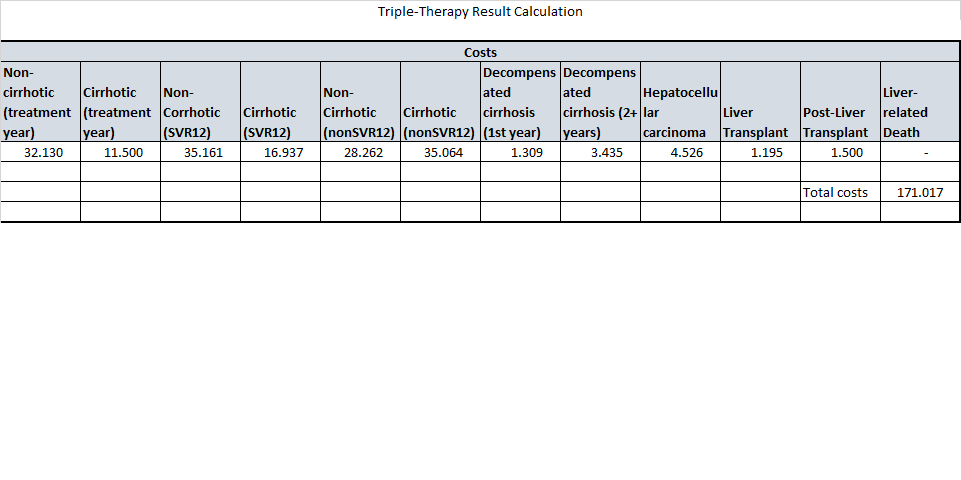

Supplement: Supplementary file 1 [file ijerph-17-00440-s001.zip › Triple Result Calculation.png]
